# AAV‐PHP.eB Peripheral Delivery and Central Expression in Cre Mice

**DOI:** 10.1002/cne.70125

**Published:** 2025-12-31

**Authors:** Haidong Zhu, Joel C. Geerling

**Affiliations:** ^1^ Department of Neurology University of Iowa Iowa City Iowa USA; ^2^ Iowa Neuroscience Institute Iowa City Iowa USA

**Keywords:** *Foxp2*, gene therapy, *Hsd11b2*, 11‐beta‐hydroxysteroid dehydrogenase type 2 (HSD2), *Lmx1b*, neuropeptide S, PHP.eB

## Abstract

Investigating the molecular and functional identity of specific neuronal populations is a critical component of neuroscience research. Specific brain regions can be targeted with stereotaxic injections of viral vectors that deliver recombinase‐dependent genetic tools, but this approach cannot target widely distributed neuronal populations. Peripheral injection is a potential strategy to access widely distributed populations, and unlike most viral vectors, PHP.eB penetrates the blood–brain barrier. However, many neurotropic viruses have tropism for specific neuronal subtypes, and it is unclear if PHP.eB transduces neurons equally across brain regions. To evaluate tropism and transduction efficiency, we examined the brain after retro‐orbital injections of a PHP.eB vector that delivers a Cre‐dependent reporter in a variety of Cre mice. We observed widely variable transduction efficiency across brain regions and cell types. Additionally, we found scattered neurons and astrocytes throughout the brain in all Cre‐driver strains, as well as Cre‐negative control mice, indicating that a low rate of spontaneous recombination had occurred. These results confirm that peripheral delivery of PHP.eB can transduce widely distributed neuronal populations. However, variations in tropism, sensitivity, and specificity merit careful consideration for each population of interest.

## Graphical abstract

Retro‐orbital injection of AAV‐PHP.eB‐FLEX‐tdTomato into four different strains of Cre mice produced various and distinct patterns of transduction efficiency and tropism across brain regions and cell types. We found many target neurons expressing tdTomato in most but not all strains, along with a low level of nonspecific (Cre‐independent) expression across brain regions in every strain and in C57BL/6J mice.

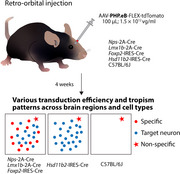

## 1 Introduction

A central challenge in neuroscience is understanding how behavior emerges from the structure and function of neural circuits. Multiple approaches have been developed to investigate neurons, and many of these use an adeno‐associated viral (AAV) vector to target specific neuronal subtypes. Typically, the AAV is delivered into a specific brain region by stereotaxic injection, but a major limitation of this approach is that transducing large neuronal populations often requires multiple injections and large amounts of AAV, making it impractical to transduce widespread populations. For both research and therapeutic applications, an approach that efficiently transduces widespread neuronal populations in the brain would represent a significant advance.

Gradinaru and colleagues developed the PHP.eB capsid, via targeted evolution of an AAV9 serotype, to transduce neurons in the brain after peripheral injection (Chan et al. 2017). This vector has been used in preclinical studies to deliver molecular tools and therapeutics to the brain (An et al. 2025; Ben‐Simon et al. 2025; Furlanis et al. 2025; Hunker et al. 2025; Neumann et al. 2024; Radhiyanti et al. 2021; Zhou et al. 2022). Although most studies to date have either targeted cells non‐selectively or used a promoter‐ or enhancer‐driven approach, using PHP.eB to deliver Cre‐dependent constructs should allow cell‐type‐specific targeting in Cre‐driver mice (Atasoy et al. 2008; Madisen et al. 2012). However, the efficacy and selectivity of this approach have not been evaluated across brain regions and neuronal subtypes.

For effective experiments and optimal therapeutics, an ideal vector would evenly transduce all target cells throughout the brain. However, it is unclear if PHP.eB transduces neurons evenly across brain regions and subpopulations, and some observations suggest that it may transduce disproportionately more cells in certain brain regions (Chan et al. 2017; Okamoto et al. 2023). Moreover, the finding that PHP.eB crosses the blood–brain barrier better in C57BL/6J mice than in certain other strains (Challis et al. 2019; Hordeaux et al. 2018; Huang et al. 2019) raises the possibility that some preclinical models may be less suitable for peripheral delivery of PHP.eB.

In this study, we evaluated the efficacy and tropism of peripheral PHP.eB delivery and Cre‐conditional transduction across brain regions and neuronal subtypes. To do this, we analyzed the brain‐wide pattern of tdTomato expression after retro‐orbital injection of a PHP.eB vector delivering Cre‐dependent tdTomato in a variety of Cre‐driver strains and in Cre‐negative control mice.

## 2 Materials and Methods

### 2.1 Mice

All mice were individually housed in a temperature‐ and humidity‐controlled room on a 12/12 h light/dark cycle, with ad libitum access to water and standard rodent chow. For retro‐orbital injections, we used *n* = 6 male and *n* = 11 female mice ranging in age from 3 to 7 weeks (7–22 g body weight) at the time of injection and 7–11 weeks (16–27 g body weight) at the time of perfusion. For stereotaxic injections, we used *n* = 4 male mice ranging in age from 7 to 8 weeks (24–25 g body weight) at the time of stereotaxic injection and 10–11 weeks (27–28 g body weight) at the time of perfusion. We used knockin‐Cre and Cre‐reporter mice, with detailed information about each strain in Table 1, in addition to C57BL/6J mice (#000664; Jackson Labs). All Cre‐driver and Cre‐reporter mice (*n* = 10) were hemizygous on a C57BL/6J background. All experiments were conducted in accordance with the guidelines of the Institutional Animal Care and Use Committee at the University of Iowa.

**TABLE 1:** Cre‐driver and ‐reporter mice.

Strain	References	Source information	Key gene
C57BL/6J	N/A	Jax 000664	N/A
*Foxp2*‐IRES‐Cre	Rousso et al. (2016)	Jax 030541	IRES‐Cre inserted after termination codon of the mouse *Foxp2* gene
*Hsd11b2*‐IRES‐Cre	Jarvie and Palmiter (2017)	Jax 030545	IRES‐Cre‐GFP inserted after the termination codon of *Hsd11b2*
*Lmx1b*‐2A‐Cre	Grady et al. (2025)	Jax 040885	2A‐Cre inserted downstream of endogenous *Lmx1b* gene
*Nps*‐2A‐Cre	Huang et al. (2022)	Jax 038113	2A‐Cre inserted downstream of the endogenous *Nps* gene
R26‐LSL‐L10GFP Reporter	Krashes et al. (2014)	Available from originating investigators	Floxed transcription STOP cassette followed by EGFP/Rpl10 fusion reporter gene under control of the CAG promoter targeted to the Gt(ROSA)26Sor locus

### 2.2 Retro‐Orbital Injections

To deliver PHP.eB, we used the retro‐orbital injection technique (Chan et al. 2017; Yardeni et al. 2011). We first anesthetized each mouse with inhaled isoflurane, then injected 100 µL of sterile 0.9% NaCl containing 1.5 × 10^11^ viral genome copies of PHP.eB‐FLEX‐tdTomato (1.5 × 10^12^ vg/mL) into the right retro‐orbital venous sinus using a 28‐gauge needle. Detailed information about this vector is provided in Table 3. Each mouse was monitored for one day post‐injection, and all mice were housed individually until they were perfused for histological analysis after 4 weeks to allow adequate time for Cre‐dependent recombination and tdTomato expression.

### 2.3 Stereotaxic Injections

*Foxp2*‐IRES‐Cre mice (*n* = 4) were anesthetized with isoflurane (0.5%–2.0%) and placed in a stereotaxic frame (Kopf; Model 1900). We made a midline incision and retracted the skin to expose bregma and the skull over the brain. Through a pulled glass micropipette (20–30 µm inner diameter), we injected 100 nL of AAV PHP.eB‐FLEX‐tdTomato (1.5 × 10^12^ vg/mL) into the ventromedial medulla, targeting the inferior olive with coordinates 6.000 mm caudal, ±0.400 mm lateral, and 6.800 mm deep to bregma. In two cases, we injected the right side, and in two cases, we injected the left side. Each injection was made over a 5‐min period using picoliter air puffs through a solenoid valve (Clippard EV 24V DC) pulsed by a Grass SD9 stimulator. The pipette was left in place for an additional 5 min, then withdrawn slowly before closing the skin with Vetbond (3M). Carprofen (5 mg/1 kg) was provided for postoperative analgesia. Mice were allowed to survive for 3 weeks after surgery to allow optimal production of Cre‐conditional proteins.

### 2.4 Perfusion and Tissue Sections

Mice were anesthetized with a mixture of ketamine‐xylazine (i.p. 150–15 mg/kg, dissolved in sterile 0.9% saline), then perfused transcardially with phosphate‐buffered saline (PBS), followed by 10% formalin‐PBS (SF100‐20, Fischer Scientific). After perfusion, each brain was removed and fixed overnight in 10% formalin‐PBS, then switched to 30% sucrose‐PBS until it sank. We sectioned each brain into 40 µm‐thick coronal slices using a freezing microtome and collected tissue sections into separate, 1‐in‐3 series. Sections were stored in cryoprotectant solution at −30°C until further processing.

### 2.5 Immunohistology

For immunofluorescence labeling, we removed the tissue sections from cryoprotectant and rinsed them in PBS before loading them into a primary antibody solution. To produce it, two or three antisera (Table 2) were added to a PBS solution containing 0.25% Triton X‐100 (BP151‐500, Fisher), 2% normal donkey serum (NDS, 017‐000‐121, Jackson ImmunoResearch), and 0.05% sodium azide (14314, Alfa Aesar) as a preservative (PBT‐NDS‐azide). We incubated tissue sections in this solution overnight at room temperature on a tissue shaker. The following morning, the sections were washed six times in PBS and incubated for 2 h at room temperature in a PBT‐NDS‐azide solution containing species‐specific donkey secondary antibodies. These secondary antibodies were conjugated to Alexa Fluor 488, Cy3, Cy5, or biotin (Jackson ImmunoResearch #s 713‐545‐003, 711‐165‐152, 706‐065‐148, 706‐545‐148, 713‐065‐147, 715‐545‐150, 711‐545‐152; each diluted 1:1000 or 1:500). If biotin was used, tissue sections were then washed again and incubated for an additional 2 h in streptavidin‐Cy5 (1:1000; SA1011; Invitrogen) in PBT‐NDS‐azide. Finally, tissue sections were washed six times in PBS, mounted on glass slides (#2575‐plus; Brain Research Laboratories), and coverslipped using Vectashield with DAPI (Vector Labs). Slides were stored in slide folders at 4°C until imaging.

**TABLE 2:** Antisera used in this study.

Antigen	Immunogen description	Source, host species, RRID	Concentration
DsRed	DsRed‐Express, a variant of *Discosoma* sp. red fluorescent protein	Clontech, rabbit polyclonal Cat# 632496 Lot# 2103116 RRID: AB_10013483	1:2000
Forkhead box protein 2 (FoxP2)	Recombinant human FoxP2 isoform 1 Ala640‐Glu715	R&D Systems, sheep polyclonal Cat# AF5647 Lot# CCUB0221031 RRID: AB_2107133	1:3000
GFP	Full‐length green fluorescent protein from the jellyfish Aequorea victoria	Invitrogen, chicken polyclonal, Cat# A10262 Lot# 2941309 RRID: AB_2534023	1:3000
11‐beta‐hydroxysteroid dehydrogenase type 2 (HSD2)	Fusion protein Ag5146 (amino acids 1–405 encoded by human HSD11B2; GenBank BC036780)	Protein Tech, rabbit polyclonal, Cat# 14192‐1‐AP Lot# 00087229 RRID: AB_2119643	1:2000
Lmx1b	Full‐length LIM homeobox transcription factor 1 beta protein from mouse	C. Birchmeier, Max Delbruck Center for Molecular Medicine, Berlin; guinea pig polyclonal RRID: AB_2314752	1:8000
Tyrosine hydroxylase (TH)	Native tyrosine hydroxylase from rat pheochromocytoma in sheep host	Millipore, sheep polyclonal Cat# AB_1542 Lot# 4048632 RRID: AB_90755	1:2000
Tyrosine hydroxylase (TH)	Tyrosine hydroxylase purified from PC12 cells	Millipore, mouse polyclonal Cat# MAB_318 Lot# 3925547 RRID: AB_2201528	1:10,000

**TABLE 3:** Viral vectors.

Injected vector	Abbreviation	Source	Origin
pAAV9‐PHP.eB.CAG.Flex.tdTomato.WPRE.SV40	PHP.eB‐FLEX‐tdTomato	Addgene Cat.# 28306‐PHPeB Lot#: v67603 (1.5 × 10^13^ vg/mL); v181392 (2.2 × 10^13^ vg/mL) RRID: Addgene_28306	CAG.Flex.tdTomato.WPRE.SV40: Edward Boyden, Massachusetts Institute of Technology PHP.eB capsid: Dr. Viviana Gradinaru, California Institute of Technology (Chan et al. 2017)

### 2.6 Microscope Imaging, Analysis, and Figures

All slides were scanned using an Olympus VS120 microscope. We began by first acquiring a 2× overview scan and then using a 10× objective to scan all tissue sections. We then acquired 20× *z*‐stacks encompassing all regions of interest for this study. For each slide, this produced a Virtual Slide Image (VSI) file containing a 10× whole‐slide layer, plus separate layers with 20× extended‐focus images in regions of interest. We used OlyVIA 4.2 (Olympus, RRID:SCR_016167) to adjust brightness and contrast and to crop and export full‐resolution images. We used Adobe Illustrator (RRID:SCR_010279) to make drawings, arrange images, and add lettering for figure layouts. Calibrated scale bars from the original OlyVIA images were traced in Illustrator to produce a white line in each figure.

## 3 Results

We examined the whole brain after peripheral injection of PHP.eB‐FLEX‐tdTomato in two or more mice from four different knockin‐Cre strains (*Nps*‐2A‐Cre; R26‐lsl‐L10GFP, *Lmx1b*‐2A‐Cre, *Foxp2*‐IRES‐Cre, and *Hsd11b2*‐IRES‐Cre). We selected these specific Cre‐driver strains based on several considerations, including convenience (they were available in our laboratory, and we are familiar with the endogenous distribution of each cell type), relevance to several fields of neuroscience, and coverage of separate and partly overlapping populations of neurons, which in aggregate span every part of the central nervous system. Additionally, due to unexpected labeling in every strain, we injected three Cre‐negative control mice. Finally, due to the conspicuous absence of tdTomato expression in the inferior olivary nucleus of *Foxp2*‐IRES‐Cre mice, we made stereotaxic injections of the same vector directly into this brainstem region in additional *Foxp2*‐IRES‐Cre mice. Below are strain‐by‐strain observations.

### 3.1 *Nps*‐2A‐Cre Mice

This strain (*Nps*‐2A‐Cre; R26‐lsl‐L10GFP, *n* = 2, injected at 24 days) had tdTomato expression in all known populations of *Nps*‐expressing neurons (Huang et al. 2022).

**FIGURE 1:**
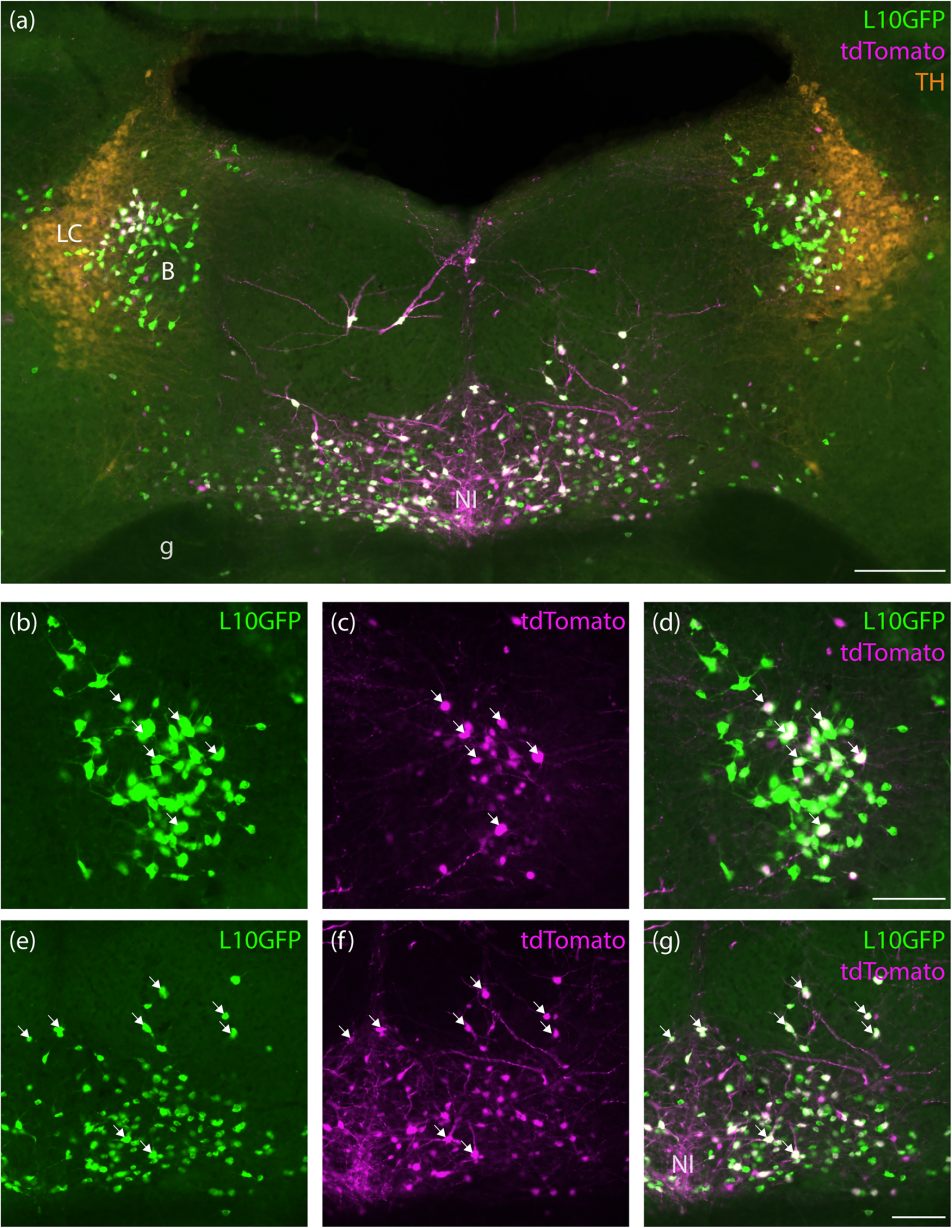
tdTomato expression in the pontine central gray matter and nucleus incertus region of an *Nps*‐2A‐Cre; R26‐LSL‐L10GFP mouse (case 8512) after retro‐orbital injection of AAV‐PHP.eB‐FLEX‐tdTomato. Neighboring the locus coeruleus (tyrosine hydroxylase, TH‐immunoreactive neurons, orange in a), we found a small population of neurons expressing the Cre‐reporter L10GFP (green, b) neurons, some of which expressed tdTomato (magenta/white, c and d). Medially, most tdTomato‐expressing neurons in the nucleus incertus (a and f) co‐expressed L10GFP (green, a, e, g). White arrows represent some examples of co‐localization of L10GFP‐ and tdTomato‐expressing neurons. Scale bar in (a) is 200 µm. Scale bars in (d) and (g) are 100 µm and apply to remaining panels in their respective rows. B, Barrington's nucleus; g, genu of facial nerve; LC, locus coeruleus; NI, nucleus incertus.

**FIGURE 2:**
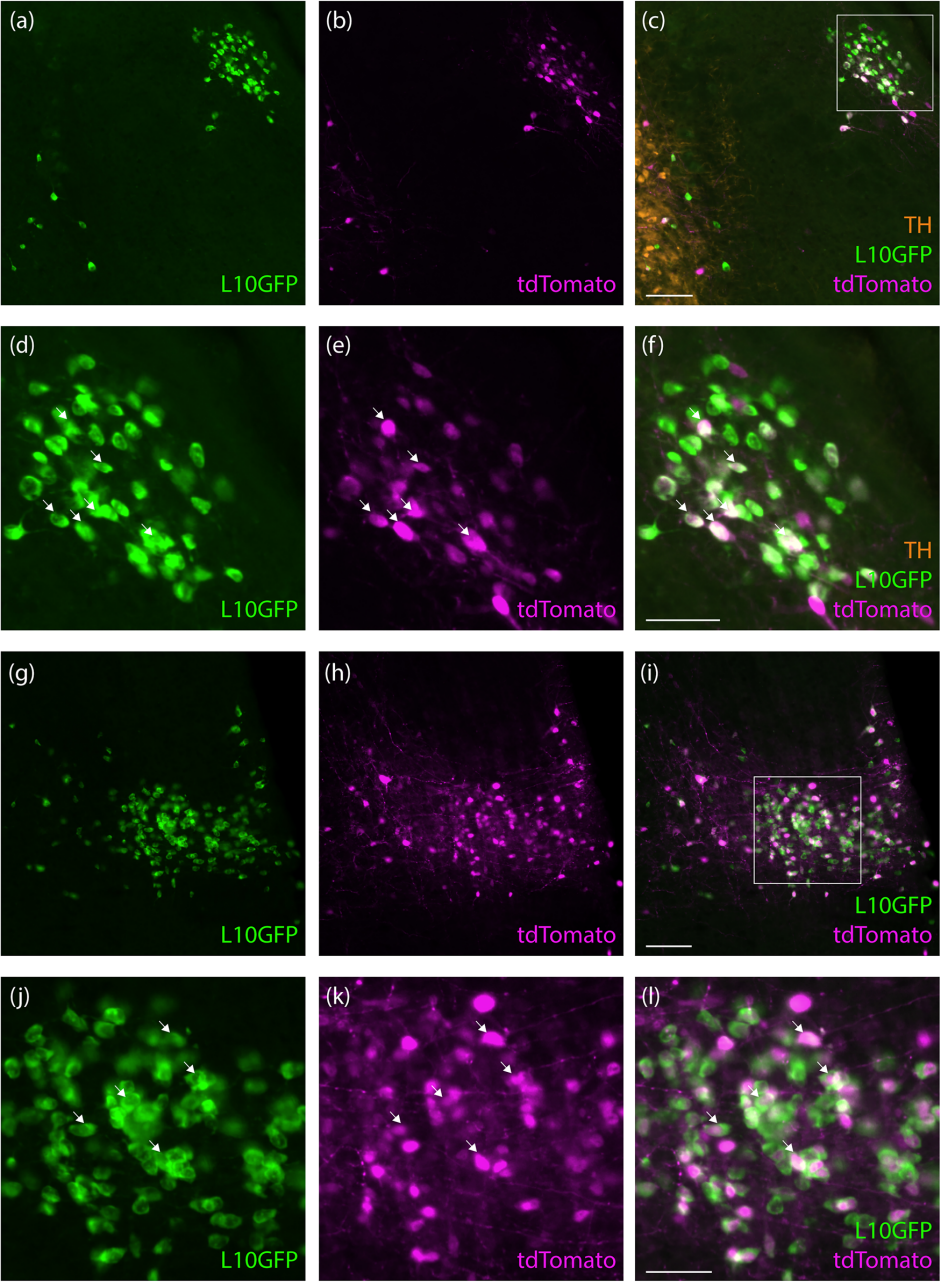
L10GFP‐ and tdTomato‐expressing neurons in the lateral parabrachial nucleus (a–f) and, further rostrally, in the “extreme lateral” parabrachial nucleus (g–l; *Nps*‐2A‐Cre case 8512). White arrows represent some examples of co‐localization of L10GFP‐ and tdTomato‐expressing neurons. Scale bars are 100 µm in panels (c) and (i), 50 µm in panels (f) and (l), and apply to remaining panels in each row.

In the brainstem, we observed a large, paramedian population of tdTomato‐expressing neurons located within the nucleus incertus and pontine central gray matter. These neurons had prominent dendrites and were distributed between the facial nerve genu on either side (Figure 1a). In this region, most L10GFP‐expressing neurons co‐expressed tdTomato (Figure 1e–g). Also, neighboring the locus coeruleus along its medial surface, we observed a small, bilateral population of L10GFP‐expressing neurons (Figure 1b–d). Many of these co‐expressed tdTomato, with more frequent co‐expression in smaller neurons located closer to the locus coeruleus relative to the sparse larger neurons located medially in Barrington's nucleus. Rostral to these, the lateral parabrachial nucleus also contained L10GFP‐ and tdTomato‐expressing neurons (Figure 2a–f), whereas the medial parabrachial nucleus contained very few, scattered neurons expressing L10GFP with and without tdTomato. Further rostrally, we found a more distinctive population at the rostral‐lateral edge of the parabrachial nucleus. Most L10GFP‐expressing neurons in this extreme‐lateral subregion co‐expressed tdTomato, and few tdTomato‐expressing neurons here lacked green fluorescence (Figure 2g–l). Immediately ventral to these neurons, the Kölliker‐Fuse nucleus did not contain any tdTomato or L10GFP, but the population of neurons in the extreme‐lateral parabrachial subregion extended rostrally, beyond the parabrachial nucleus, wrapping around the nucleus of the lateral lemniscus and through neighboring regions that include the semilunar and paralemniscal nuclei (Gomez‐Martinez et al. 2023).

**FIGURE 3:**
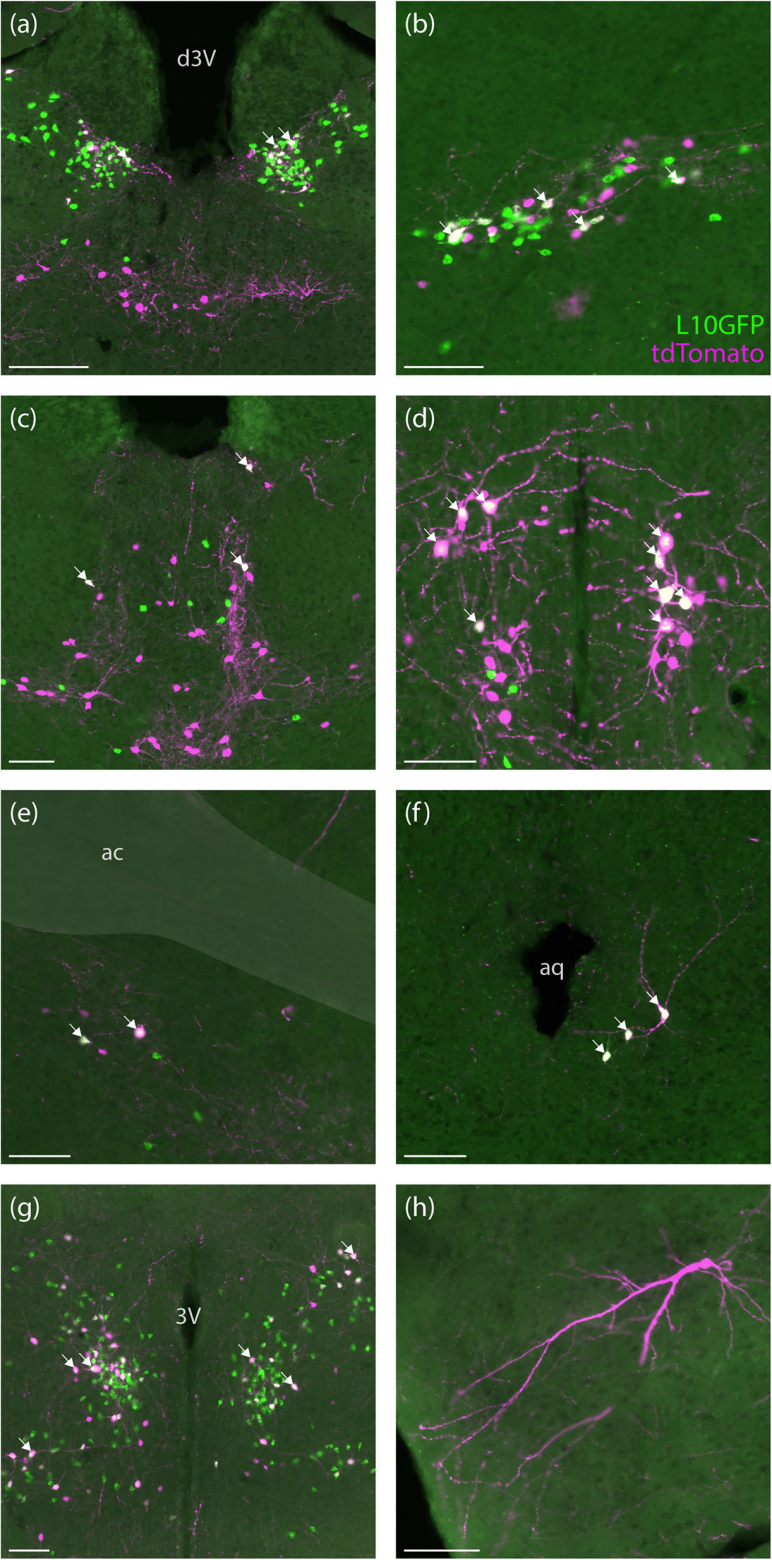
*Nps*‐2A‐Cre; R26‐LSL‐L10GFP cases (case 8512) had small populations of L10GFP‐expressing (green) and tdTomato‐expressing (magenta) neurons in the lateral habenula and paraventricular thalamic nucleus (a), pretectal region (b), anterior paraventricular thalamic nucleus (c), rostral periaqueductal gray matter at the midbrain–diencephalic junction (d), anterior ventral bed nucleus of the stria terminalis (e), ventrolateral periaqueductal gray matter (f), and anterior hypothalamus (g). Additionally, few sparse tdTomato‐expressing neurons were found in the nucleus of stria terminalis (e) and periaqueductal gray (f). Each case also had sparse tdTomato‐expressing pyramidal neurons at every level of the cerebral cortex (h). White arrows represent some examples of co‐localization of L10GFP‐ and tdTomato‐expressing neurons. All scale bars are 100 µm. 3V, third ventricle; ac, anterior commissure; aq, cerebral aqueduct; d3V, dorsal third ventricle.

In the forebrain, we identified clusters of tdTomato‐expressing neurons in the lateral habenula, paraventricular nucleus of the thalamus, and anterior hypothalamus. Immediately ventrolateral to the medial habenula border, a discrete ventral subpopulation of neurons in the lateral habenula expressed L10GFP, and a minority of these co‐expressed tdTomato (Figure 3a). Caudal to these, we noted another small population of neurons in the pretectal region (Figure 3b). In and alongside the paraventricular thalamic nucleus, we found a modest number of tdTomato‐expressing neurons, many of which lacked L10GFP (Figure 3a,c). At the midbrain–diencephalic junction, we observed a small number of co‐expressing neurons, along with tdTomato‐expressing neurons that flanked the dorsal third ventricle, just medial to the A11 catecholamine neurons (Figure 3d). The anterior hypothalamus near the paraventricular hypothalamic nucleus contained a separate population of tdTomato‐ and L10GFP‐co‐expressing neurons (Figure 3g). We also noted sparse tdTomato‐expressing neurons in the ventral bed nucleus of the stria terminalis (Figure 3e), periaqueductal gray (Figure 3f), and medial amygdala (not shown); most of these co‐expressed L10GFP.

In addition to tdTomato co‐expression in these previously characterized regions with *Nps* Cre‐reporter expression, both mice had sparse neurons and astrocytes at every level of the cerebral cortex and in the cerebellar cortex, and none of these co‐localized with L10GFP (Figure 3h).

### 3.2 *Lmx1b*‐2A‐Cre Mice

In this strain (*Lmx1b*‐2A‐Cre, *n* = 2, injected at 20 and 36 days), tdTomato expression largely resembled the established pattern of *Lmx1b* expression in postnatal mice (Asbreuk et al. 2002; Dai et al. 2008; Zou et al. 2009) but with variable efficiency of labeling across regions. The pattern outlined below was highly similar in both mice.

**FIGURE 4:**
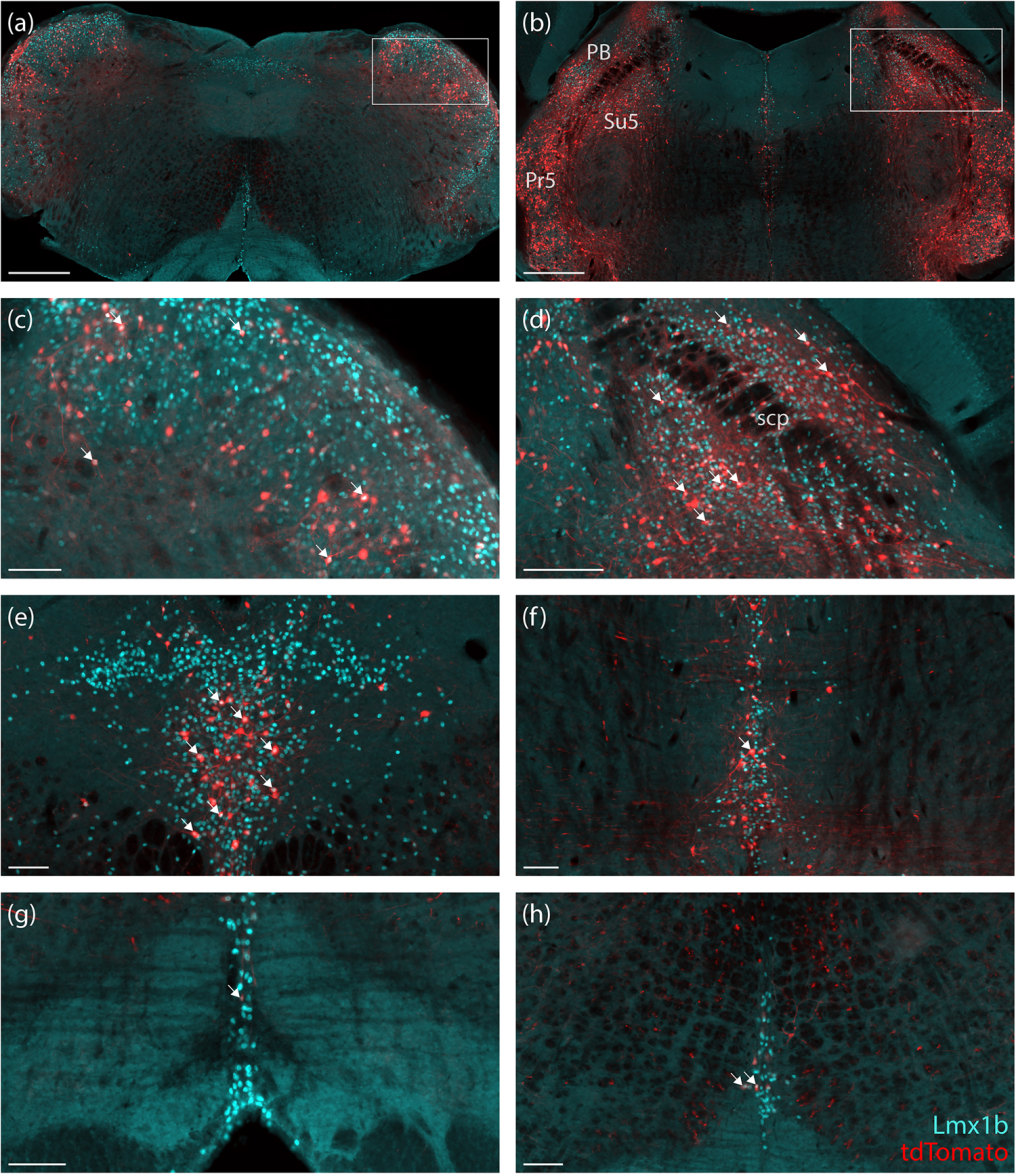
In *Lmx1b‐*2A‐Cre mice (case 8535), the caudal spinal trigeminal nucleus included a large population of tdTomato‐expressing neurons (a and c). (b) The principal sensory trigeminal, supratrigeminal, and parabrachial nuclei (d) also contained prominent populations of tdTomato‐expressing (red) neurons with co‐localized nuclear Lmx1b‐immoreactivity (ice blue). Smaller subsets of Lmx1b‐immunoreactive neurons expressed tdTomato in the dorsal raphe nucleus (e) and central superior (median) raphe nucleus (f), and fewer neurons expressed tdTomato in the raphe pallidus (g) and obscurus (h). White arrows represent some examples of co‐localization between tdTomato and Lmx1b nuclear immunoreactivity. Scale bars are 500 µm in (a and b), 100 µm in (c), 200 µm in (d), and 100 µm in (e–h). PB, parabrachial nucleus; Pr5, principal sensory trigeminal nucleus; scp, superior cerebellar peduncle; Su5, supratrigeminal nucleus.

**FIGURE 5:**
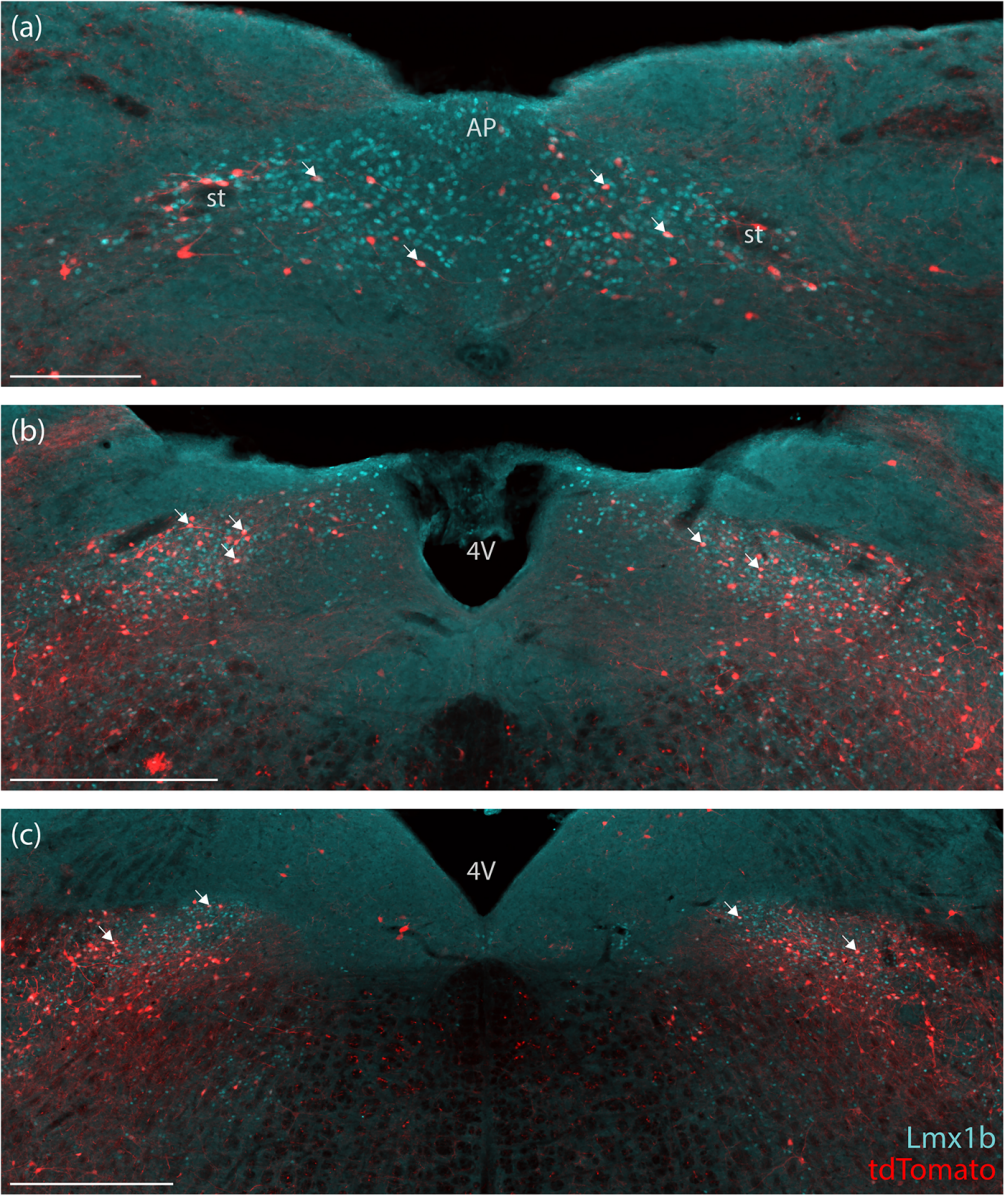
In the caudal nucleus of the solitary tract and area postrema of *Lmx1b*‐2A‐Cre mice (a), case 8535), a small minority of Lmx1b‐immunoreactive neurons (ice blue) expressed tdTomato (red). Intermediate (b) and rostral (c) levels of the nucleus of the solitary tract contained more tdTomato co‐expressing neurons. White arrows represent some examples of co‐localization between tdTomato and Lmx1b nuclear immunoreactivity. Scale bar is 200 µm in (a) and 500 µm for (b and c). 4V, fourth ventricle; AP, area postrema; st, solitary tract.

In the hindbrain, we found many tdTomato‐expressing, Lmx1b‐immunoreactive neurons in the spinal trigeminal nucleus (Figure 4a,c), increasing in density caudally through the medullary and cervical dorsal horn (not shown). Rostrally, we identified prominent labeling in the principal sensory trigeminal and supratrigeminal nuclei (Figure 4b), and dorsal to these, many Lmx1b‐immunoreactive neurons in the parabrachial nucleus expressed tdTomato (Figure 4b,d). In the nucleus of the solitary tract and area postrema, many glutamatergic neurons express *Lmx1b* (Gasparini, Almeida‐Pereira, et al. 2024), yet only a minority of Lmx1b‐immunoreactive neurons in the caudal nucleus of the solitary tract and area postrema expressed tdTomato (Figure 5a). At successively rostral levels, tdTomato‐expressing neurons formed an increasing proportion of Lmx1b‐immunoreactive neurons in the lateral nucleus of the solitary tract and extended ventrolaterally into the reticular formation (Figure 5b,c).

The most prominent Lmx1b‐immunoreactivity in the brainstem runs down the midline, spanning all raphe nuclei. From the midbrain down through the ventral hindbrain, every raphe nucleus had sparse tdTomato expression. Each tdTomato‐expressing raphe neuron contained an Lmx1b‐immunoreactive nucleus, but these neurons represented a small subset of Lmx1b‐immunoreactive neurons in the dorsal and median (central superior) raphe nuclei (Figure 4e,f), and the medullary raphe nuclei contained even fewer (Figure 4g,h).

**FIGURE 6:**
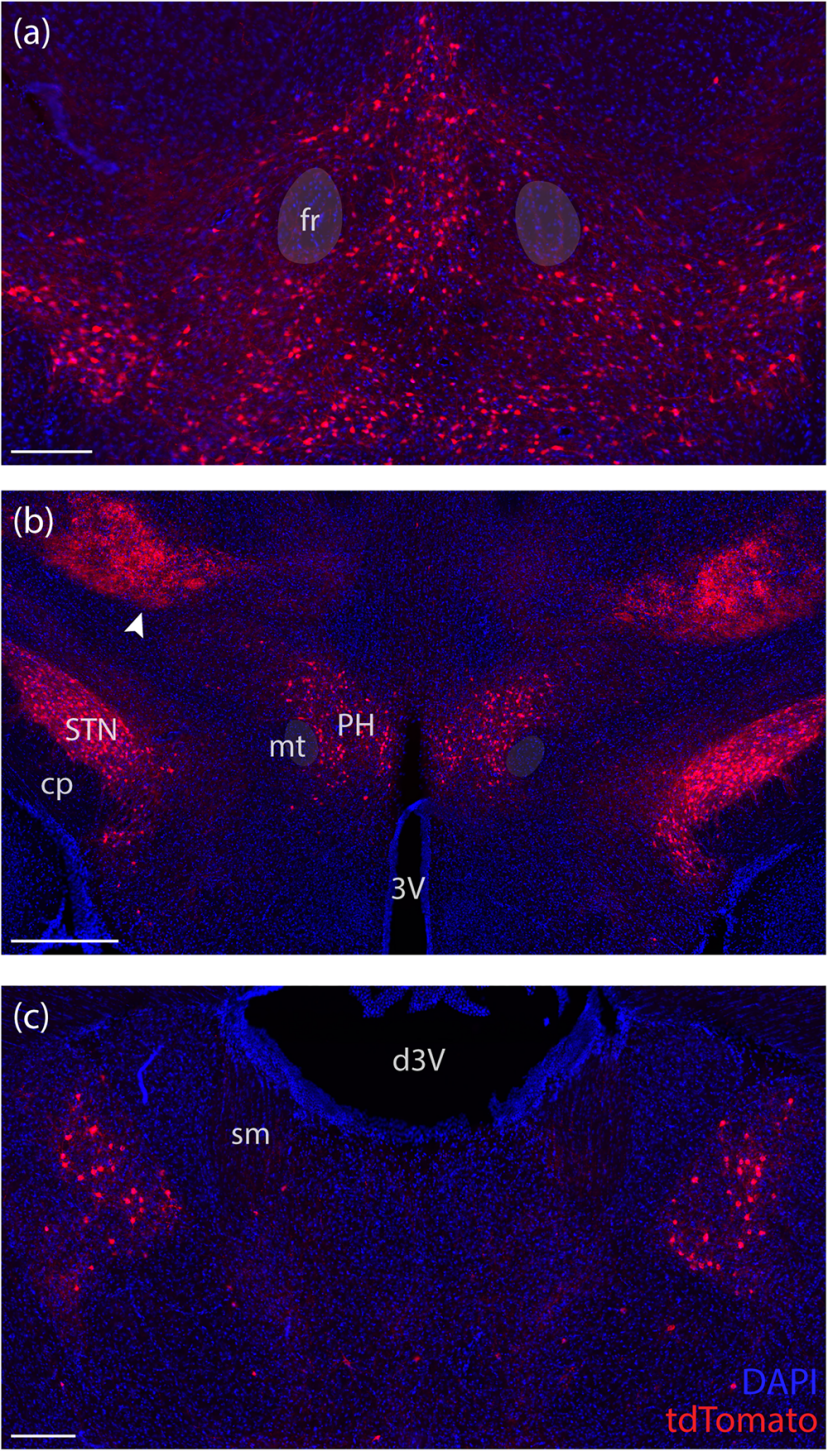
In the ventral midbrain, *Lmx1b*‐2A‐Cre cases had scattered tdTomato‐expressing neurons (red) in the ventral tegmental area (a). The posterior hypothalamus (b) included prominent collections of tdTomato‐expressing neurons in the subthalamic (STN), parasubthalamic, and posterior hypothalamic (PH) nuclei. Arrowhead indicates the bilaterally dense field of tdTomato varicose axons in the ventral posterior medial thalamic nucleus. (c) We also found tdTomato‐expressing neurons in the anteroventral thalamic nucleus (case 8764). Scale bars are 200 µm in (a and c) and 500 µm in (b). 3V, third ventricle; cp, cerebral peduncle; d3V, dorsal third ventricle; fr, fasciculus retroflexus; mt, mammillothalamic tract; sm, stria medullaris.

Further rostrally, we found scattered tdTomato‐expressing neurons in the ventral tegmental area (Figure 6a). In the hypothalamus, we observed distinctive populations of tdTomato‐expressing neurons in the subthalamic, parasubthalamic, and posterior hypothalamic nuclei (Figure 6b), as well as the ventral premammillary and supramammillary nuclei (not shown), all of which contain populations of Lmx1b‐expressing neurons (Asbreuk et al. 2002; Dai et al. 2008). Rostral to this posterior region, we did not find any other collections of tdTomato‐expressing neurons in the hypothalamus.

In the thalamus, which lacks *Lmx1b* expression (Dai et al. 2008), we noted a small population of tdTomato‐expressing neurons (Figure 6c). Also, similar to the *Nps*‐2A‐Cre cases described above, both *Lmx1b*‐2A‐Cre cases had scattered tdTomato‐expressing neurons and astrocytes at every level of the cerebral and cerebellar cortex (not shown), neither of which contain any *Lmx1b*‐expressing cells (Asbreuk et al. 2002; Dai et al. 2008).

### 3.3 *Foxp2*‐IRES‐Cre Mice

In this strain (*Foxp2*‐IRES‐Cre, *n* = 4, one injected at 42 days and three at 28 days), we observed tdTomato expression in most regions with *Foxp2*‐expressing neurons (Ferland et al. 2003; Karthik et al. 2022; Kast et al. 2019; Sarropoulos et al. 2021; Tasic et al. 2018), including the cerebral cortex, cerebellum, inferior colliculus, parabrachial nucleus, thalamus, basal ganglia, and amygdala.

**FIGURE 7:**
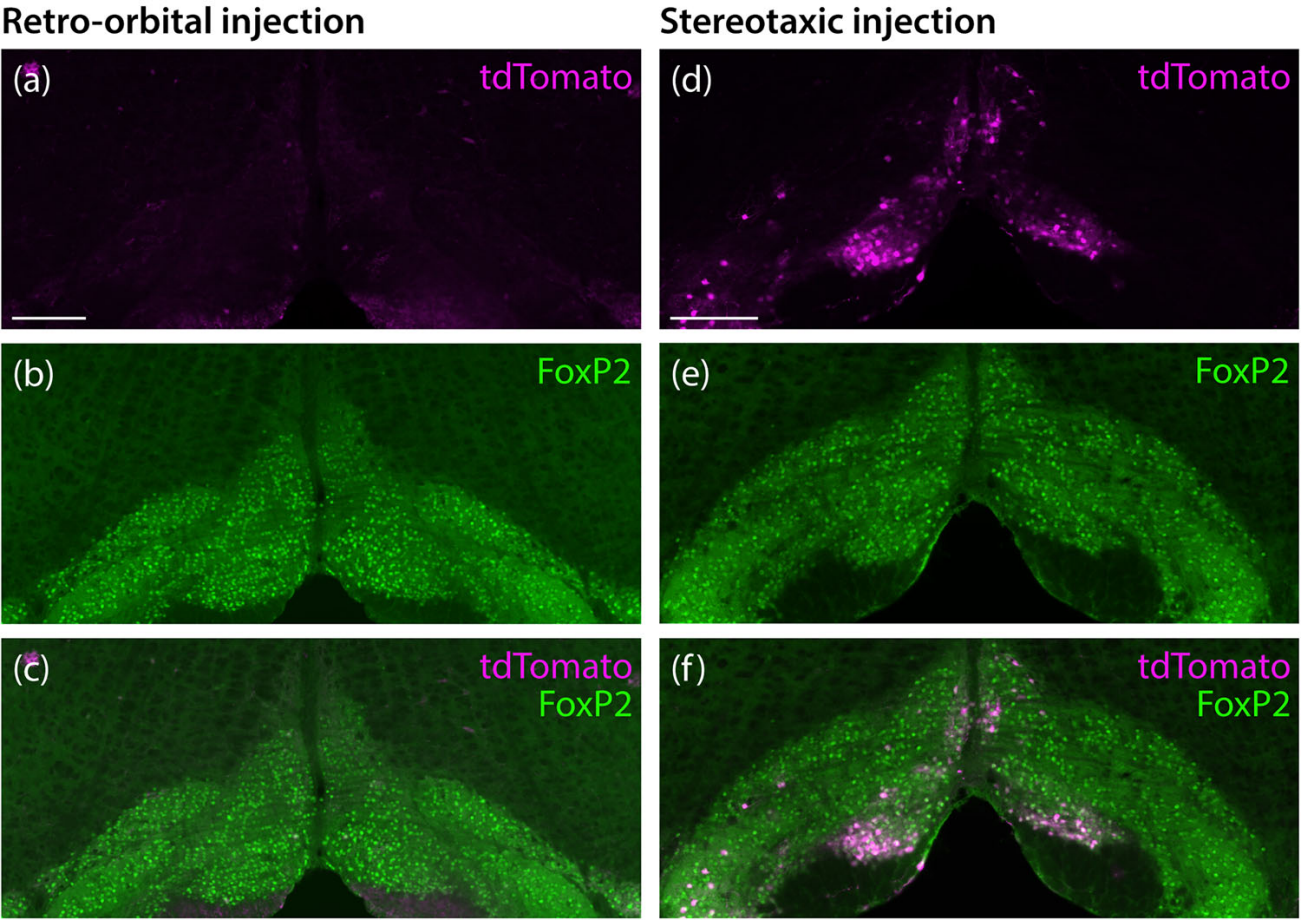
Retro‐orbital injection of PHP.eB‐Flex‐tdTomato in *Foxp2*‐IRES‐Cre mice (a–c) produced very few tdTomato‐expressing neurons (magenta) in the inferior olivary nucleus, whose neurons contain prominent FoxP2 nuclear immunoreactivity (green). Direct, stereotaxic injection of AAV‐PHP.eB‐FLEX‐tdTomato into the ventral medulla produced more tdTomato‐expressing, FoxP2‐immunoreactive neurons in the inferior olivary nucleus (d–f). Scale bars in (a) and (d) are 200 µm and apply to all panels in each respective column.

However, in the most prominent *Foxp2*‐expressing site in the brainstem, the inferior olivary nucleus, we found very little tdTomato (Figure 7a–c). Most tissue sections through the inferior olive contained just one or two tdTomato‐expressing neurons, and some contained zero. This surprising lack of transduction could be due to inability of PHP.eB to reach or transduce neurons in this location or to relative inability of inferior olivary neurons to recombine or express the Cre‐dependent tdTomato construct. To test whether AAV‐PHP.eB is capable of transducing olivary neurons, we stereotaxically injected the same vector directly into the inferior olivary nucleus in additional *Foxp2*‐IRES‐Cre mice (*n* = 4). Mice with on‐target injections (*n* = 2) had many more tdTomato‐expressing, FoxP2‐immunoreactive neurons in the inferior olivary nucleus (Figure 7d–f), along with tdTomato labeling of climbing fibers in the contralateral cerebellar cortex (not shown).

**FIGURE 8:**
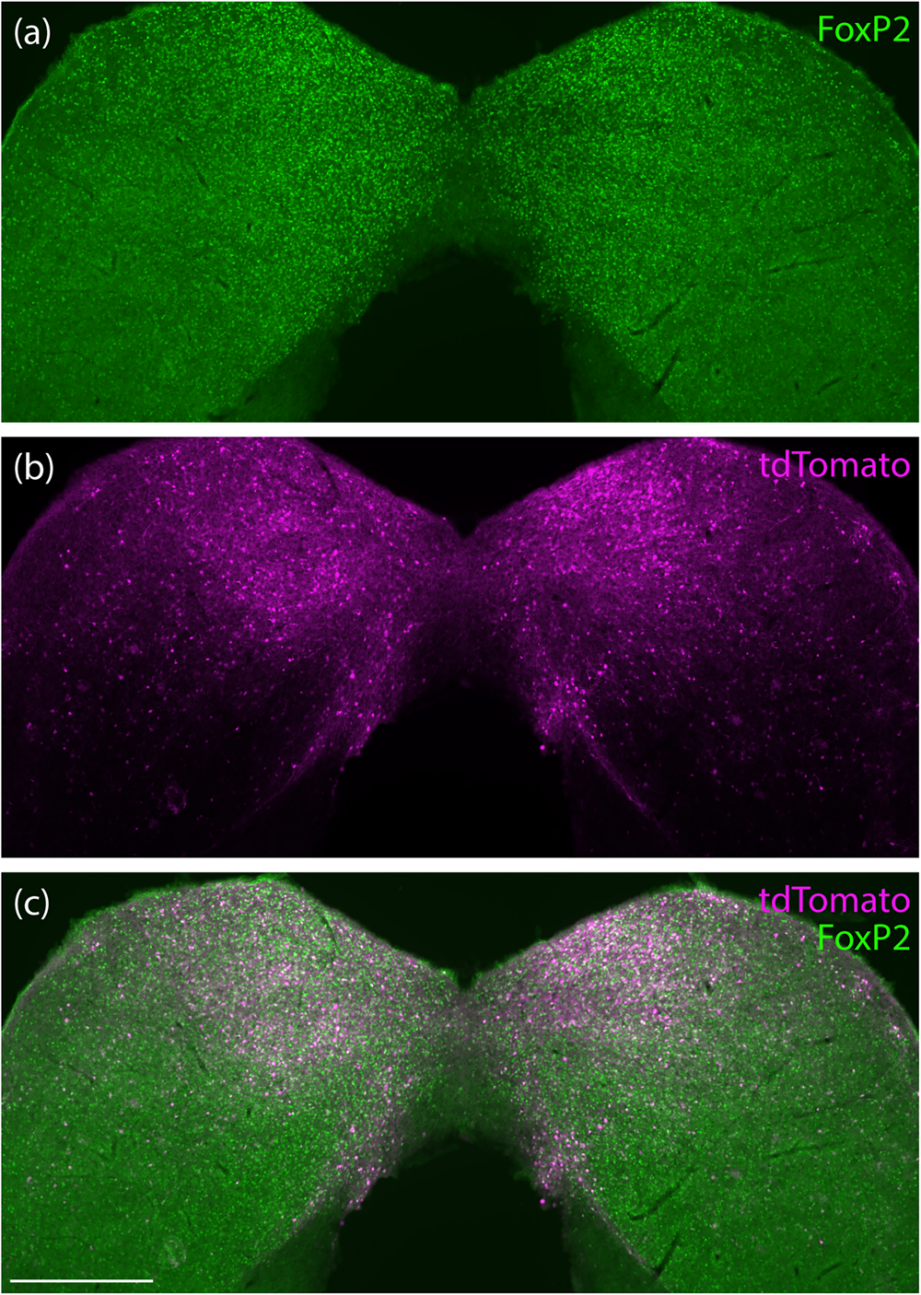
(a–c) In *Foxp2*‐IRES‐Cre mice, a dorsomedial subregion of the inferior colliculus contained many Foxp2‐immunoreactive neurons (green) that co‐expressed tdTomato (magenta; case 9035). Scale bar in (c) is 500 µm and applies to all remaining panels.

**FIGURE 9:**
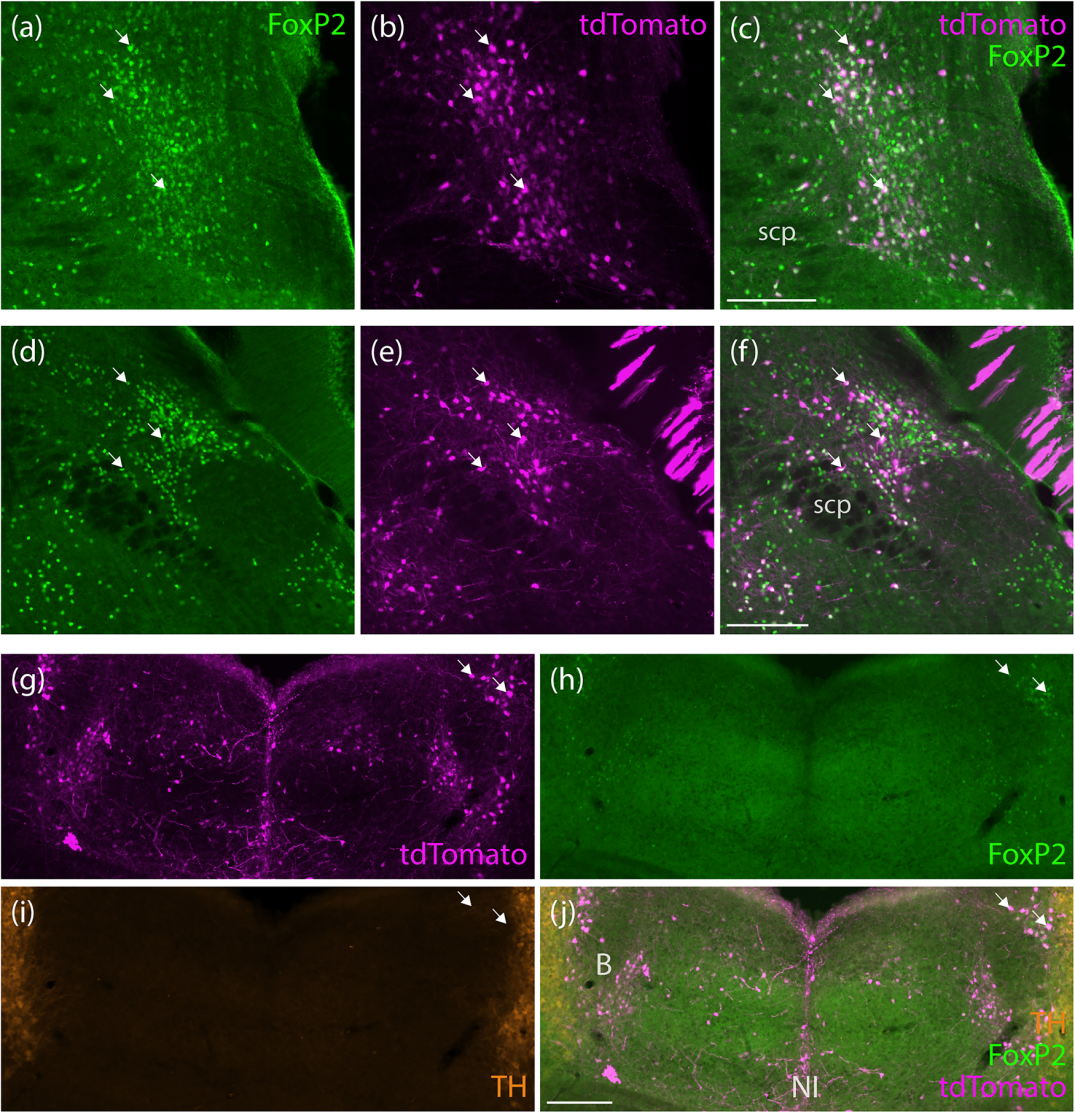
In *Foxp2*‐IRES‐Cre mice, rostral (a–c) and middle (d–f) levels of the parabrachial nucleus contained many tdTomato‐expressing neurons (magenta) with nuclear FoxP2‐immunoreactivity (green). We also found tdTomato and FoxP2 co‐expressing neurons medial to and surrounding Barrington's nucleus (B), and additional tdTomato‐expressing neurons in the nucleus incertus without FoxP2 (*Foxp2*‐IRES‐Cre case 9035). White arrows represent some examples of co‐localization of tdTomato and FoxP2 nuclear immunoreactivity. Scale bars are 200 µm for (g–j) and 100 µm for (a–f). NI, nucleus incertus; scp, superior cerebellar peduncle.

Outside the inferior olive, other *Foxp2*‐expressing brainstem regions had more tdTomato expression. For example, many FoxP2‐immunoreactive neurons in the dorsomedial inferior colliculus expressed tdTomato (Figure 8a–c). In the lateral parabrachial nucleus, we identified many tdTomato‐expressing FoxP2‐immunoreactive neurons (Figure 9a–f). Also, tdTomato co‐localized in some FoxP2‐immunoreactive neurons surrounding Barrington's nucleus (Verstegen et al. 2017), and we noted tdTomato‐expressing neurons in the nucleus incertus, but these had no discernable FoxP2 immunoreactivity (Figure 9g–j).

**FIGURE 10:**
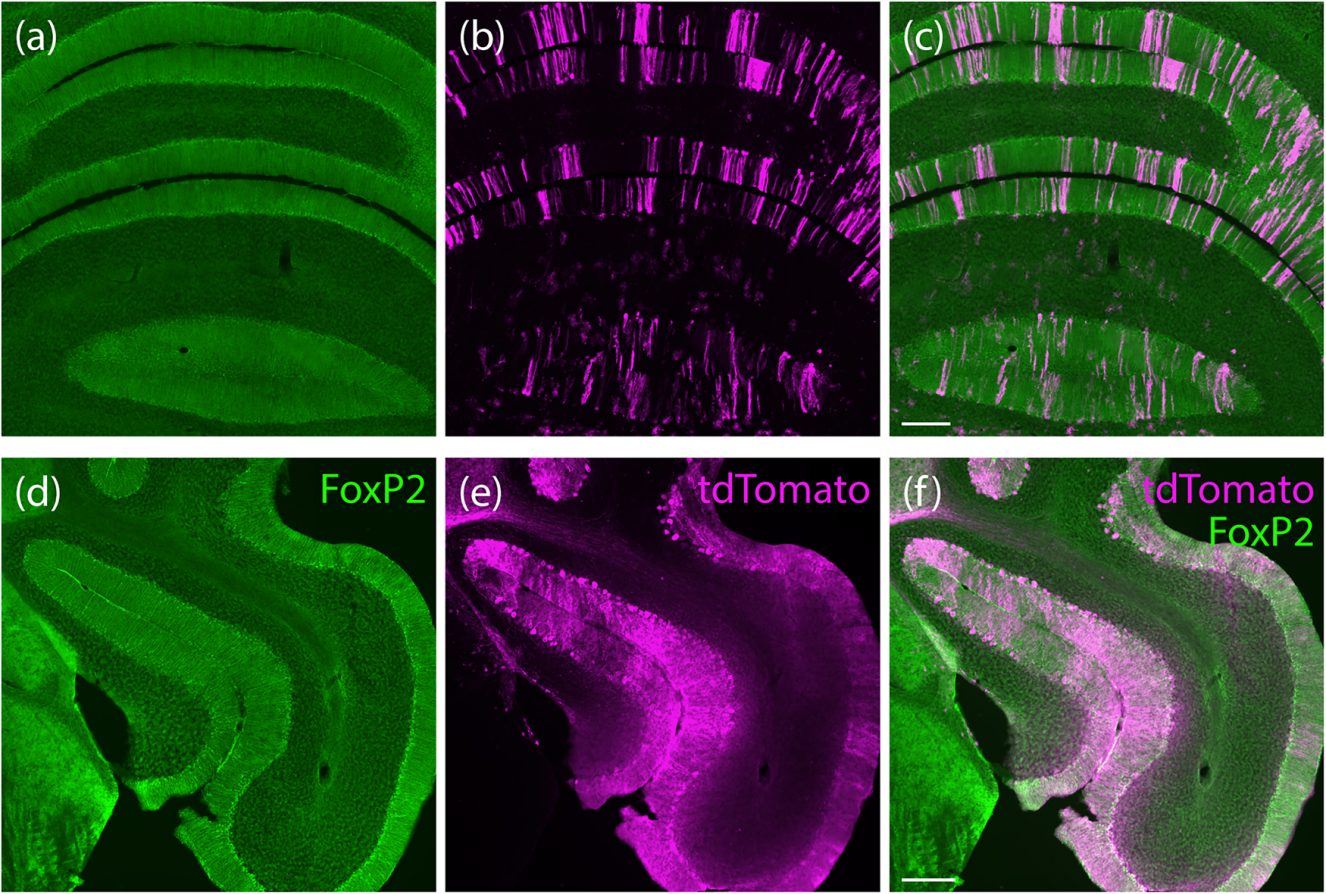
tdTomato expression in the cerebellum of *Foxp2*‐IRES‐Cre cases. We found an irregular, striped pattern of tdTomato expression in cerebellar Purkinje neurons, with tdTomato filling their dendritic arbors in the cerebellar molecular layer (a–c). The cerebellar flocculus had a higher density of tdTomato‐expressing Purkinje neurons (d–f; *Foxp2*‐IRES‐Cre case 9035). Scale bars in (c) and (f) are 200 µm and apply to remaining panels in each row.

In the cerebellum, we observed light nuclear immunoreactivity for FoxP2 throughout the Purkinje layer, consistent with previous evidence that Purkinje neurons express *Foxp2* (Ferland et al. 2003; Sarropoulos et al. 2021). Among these neurons, we observed an irregular, striped pattern of tdTomato in a minority of Purkinje cells and their distinctive dendritic arbors, which extend into the molecular layer (Figure 10a–c). Most Purkinje neurons were unlabeled, even in the flocculus, which had the highest labeling density in the cerebellum (Figure 10d–f). Nevertheless, *Foxp2*‐IRES‐Cre cases had substantially more tdTomato expression in the cerebellum than any other cases in our study.

**FIGURE 11:**
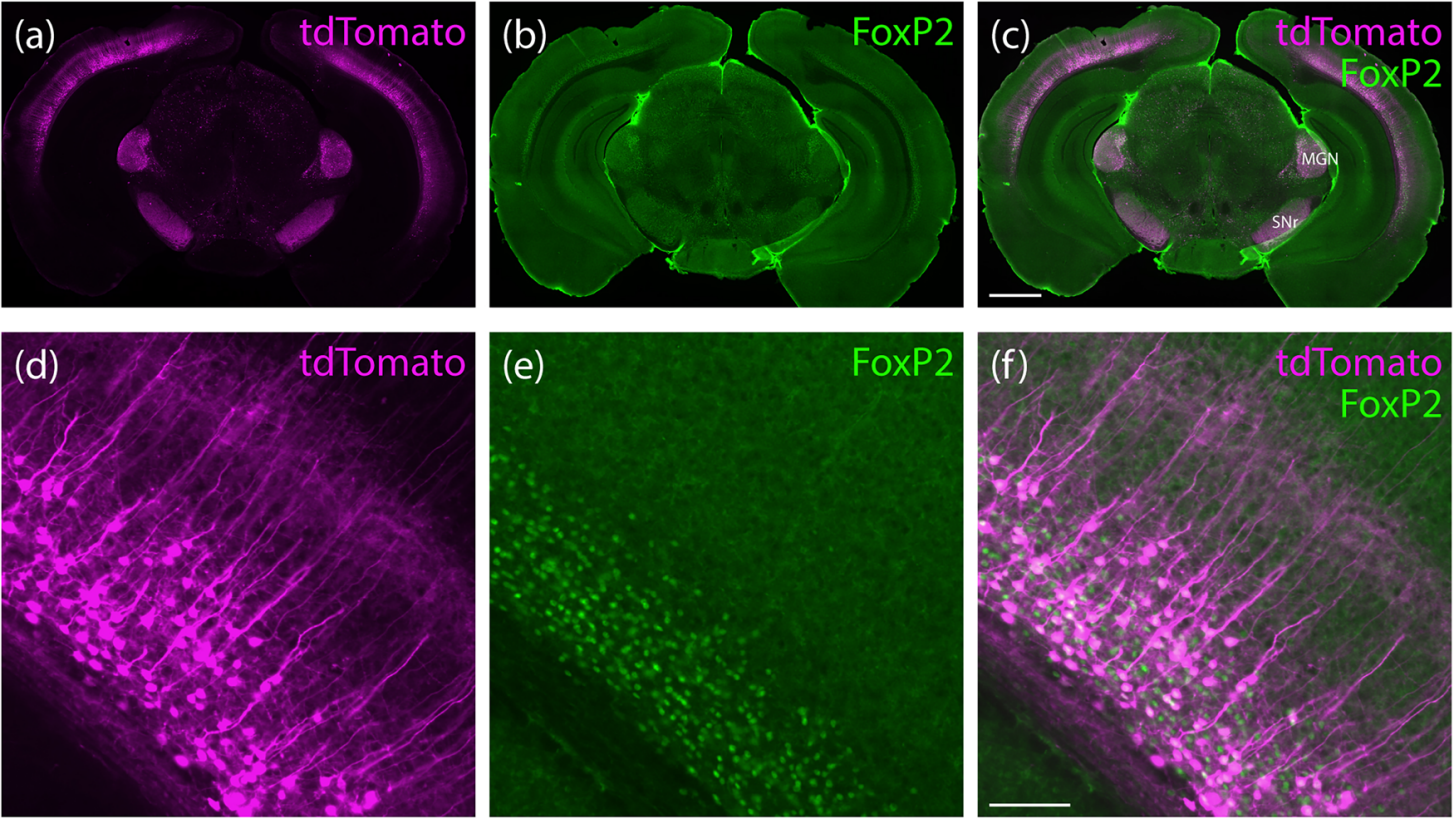
tdTomato expression in the cerebral cortex of *Foxp2*‐IRES‐Cre cases. Many cortical layer 6 neurons expressed tdTomato (magenta, a), co‐localized with FoxP2 nuclear immunoreactivity (green, b and c). At this caudal, midbrain–diencephalic level, axonal tdTomato and tdTomato‐expressing neurons were also present in and around the medial geniculate nucleus (MGN), and tdTomato axonal labeling was prominent in the substantia nigra pars reticulata (SNr). Panels (d–f) show a magnified view of cortical layer 6 in the brain tissue section shown in panels (a–c; *Foxp2*‐IRES‐Cre case 8473). Scale bars are 1 mm for (a–c) and 100 µm for (d–f).

In the forebrain, we found a conspicuous band of tdTomato‐expressing neurons throughout layer 6 of the neocortex (Figure 11a–f). Consistent with previous evidence that a majority of layer‐6 neurons express *Foxp2* mRNA and have nuclear immunoreactivity for FoxP2 (Kast et al. 2019; Tasic et al. 2018), roughly half expressed tdTomato at every rostrocaudal level, in both cerebral hemispheres, vastly exceeding the light and scattered tdTomato expression in the cerebral cortex of other strains.

**FIGURE 12:**
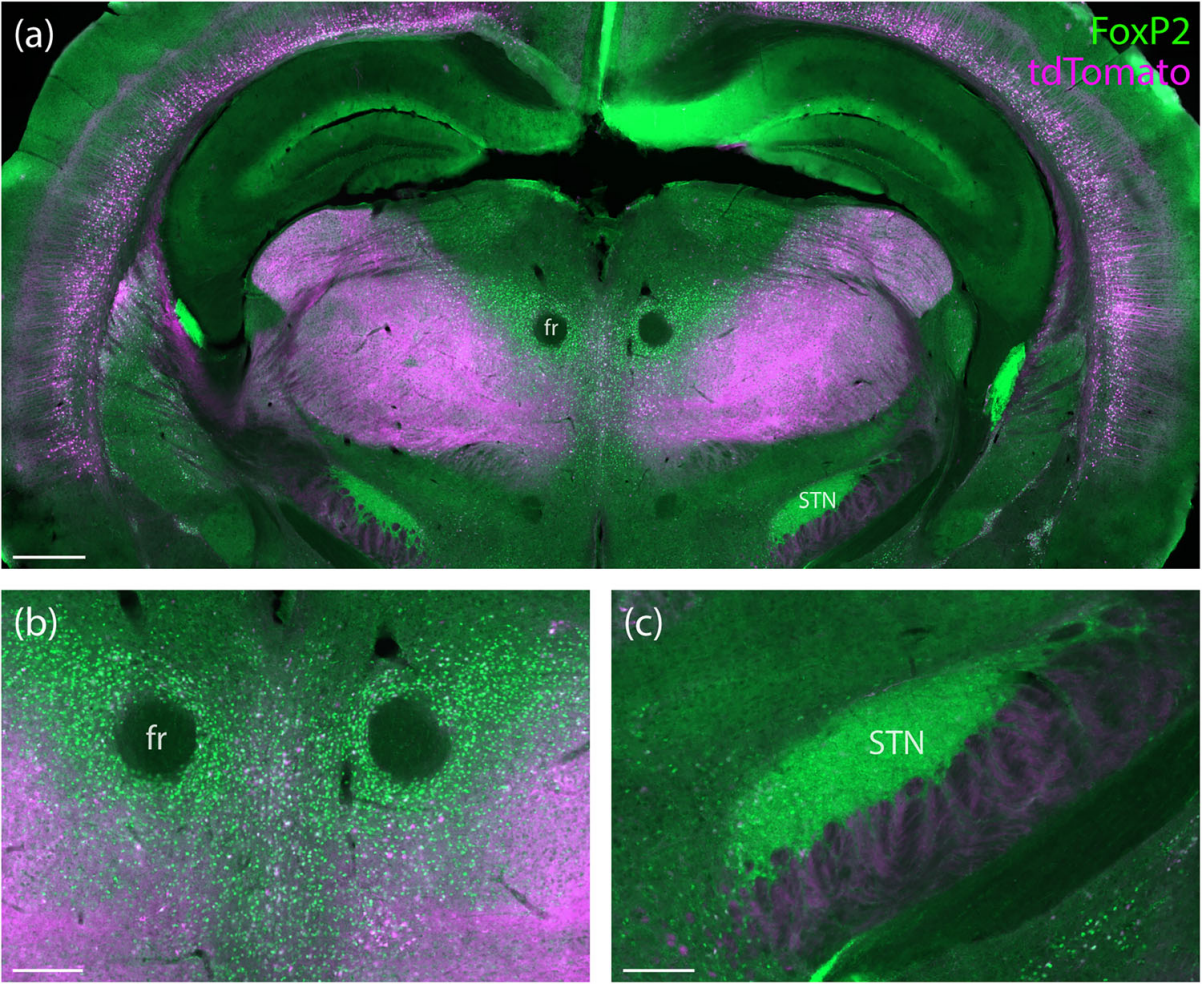
The posterior diencephalon of *Foxp2*‐IRES‐Cre cases contained abundant axonal tdTomato (a; magenta). We found very little tdTomato expression in the prominently FoxP2‐immunoreactive neurons of the parafasicular (b) and subthalamic (c) nuclei (*Foxp2*‐IRES‐Cre case 9035). Scale bars are 500 µm in (a) and 200 µm in (b) and (c). fr, fasciculus retroflexus; STN, subthalamic nucleus.

Elsewhere in the forebrain, we observed a complex pattern of tdTomato expression in *Foxp2*‐expressing populations spanning the basal ganglia, diencephalon, and amygdala (not shown). In most of these populations, a broad and substantial minority of FoxP2‐immunoreactive neurons expressed tdTomato, but in contrast, very few neurons expressed tdTomato in the prominent FoxP2‐immunoreactive populations of the subthalamic and parafascicular thalamic nuclei (Figure 12a,b,c).

Similar to *Nps*‐2A‐Cre and *Lmx1b*‐2A‐Cre cases, all *Foxp2*‐IRES‐Cre mice had labeling in scattered astrocytes across the brain. In *Foxp2*‐IRES‐Cre cases, abundant labeling in neocortical layer 6 and cerebellar Purkinje neurons prevents us from concluding that cortical or cerebellar labeling was necessarily nonspecific, but these cases also had sparse cortical tdTomato expression outside layer 6, in neurons and astrocytes that lacked FoxP2 immunoreactivity. Additionally, of the four *Foxp2*‐IRES‐Cre injected peripherally with PHP.eB, three had highly similar patterns of tdTomato expression, but the fourth had sparse labeling in the same overall pattern, plus a similar pattern of nonspecific, sparse neuronal and astrocytic labeling in the cerebral cortex.

### 3.4 *Hsd11b2*‐IRES‐Cre Mice

**FIGURE 13:**
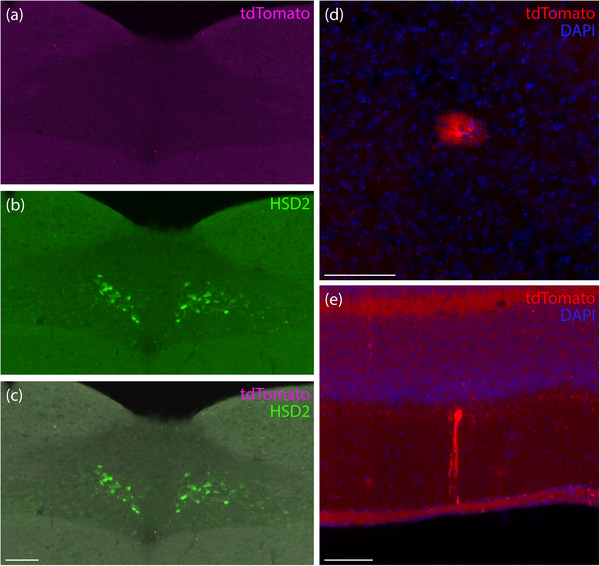
(a–c) *Hsd11b2*‐IRES‐Cre mice had no tdTomato expression (magenta) in the HSD2 (11‐beta‐hydroxysteroid dehydrogenase type 2, green) neurons of the nucleus of solitary tract (case 8807). Similar to other strains, these mice had nonspecific tdTomato expression in scattered astrocytes (d), cortical pyramidal neurons, and cerebellar Purkinje neurons (e) throughout the brain (*Hsd11b2*‐IRES‐Cre case 8464). Scale bars are 100 µm (c) and 50 µm (d and e).

None of the mice from this strain (*Hsd11b2*‐IRES‐Cre, *n* = 4, two injected at 48 days and two at 29 days) had any tdTomato‐expressing neurons in the nucleus of the solitary tract (Figure 13a–c). Immunolabeling the enzyme 11‐beta‐hydroxysteroid dehydrogenase type 2 (HSD2) identified the expected distribution of aldosterone‐sensitive neurons that are unique to this region (Gasparini, Almeida‐Pereira, et al. 2024; Gasparini, Peltekian, et al. 2024; Gasparini et al. 2019; Jarvie and Palmiter 2017; Resch et al. 2017), but none of these neurons co‐expressed tdTomato.

Although none of these mice had prominent tdTomato expression anywhere in the brain, *Hsd11b2*‐IRES‐Cre mice had labeling in scattered astrocytes, cortical pyramidal neurons, and cerebellar Purkinje neurons, similar to the other Cre‐driver strains we tested (Figure 13d–e). Unlike the other three strains described above, this sparse and nonspecific labeling was the only tdTomato expression we found in *Hsd11b2*‐IRES‐Cre cases.

### 3.5 Cre‐Negative Control Mice

**FIGURE 14:**
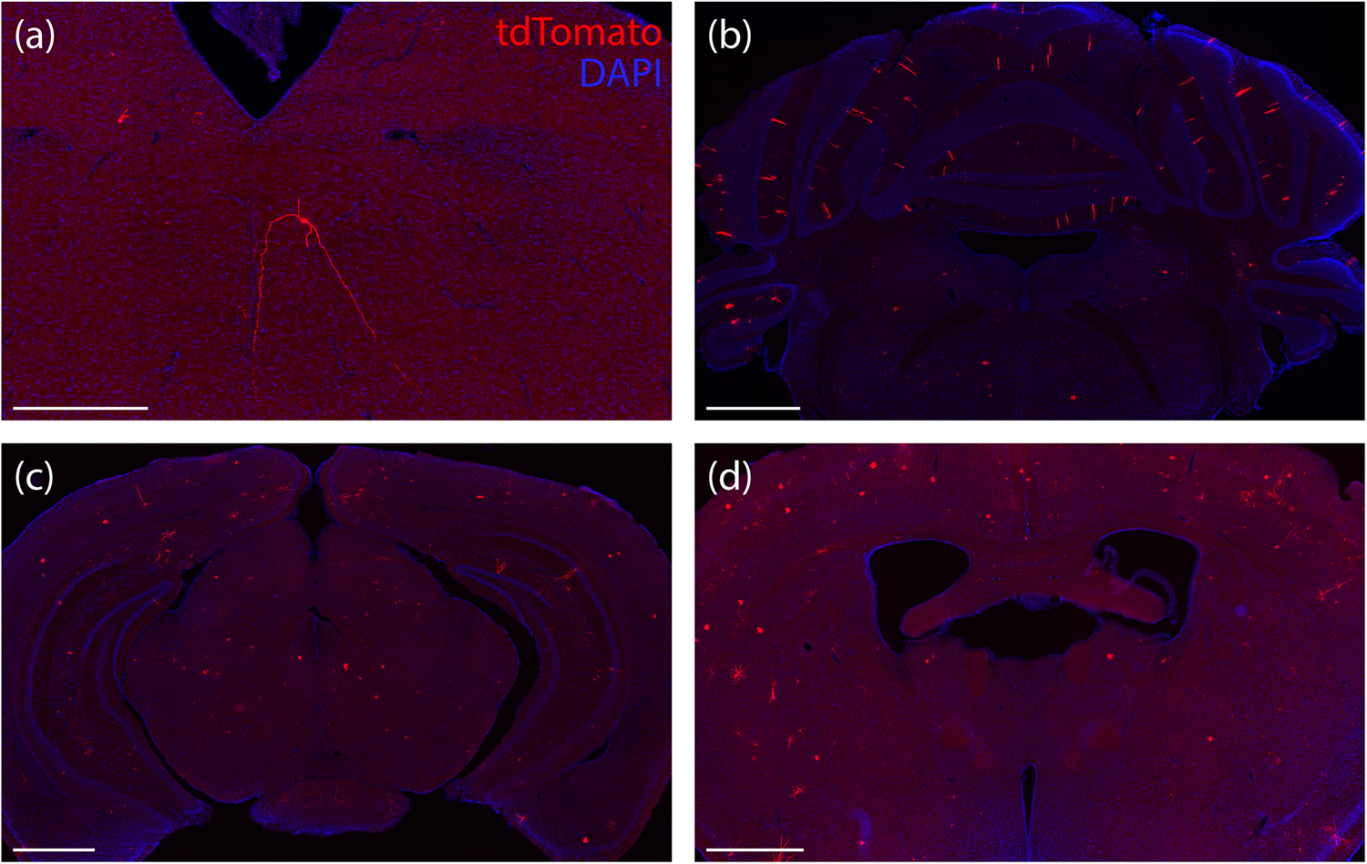
Cre‐negative control mice injected with PHP.eB‐FLEX‐tdTomato all had scattered tdTomato expression across the brains (C57BL6/J cases 8558 and 8842): (a) Isolated tdTomato‐expressing neuron and dendritic arbor in the reticular formation of the rostral medulla; (b) Scattered Purkinje neurons and their dendritic arbors in the cerebellum; (c) midbrain–diencephalic junction; (d) rostral forebrain level. Scale bars are 500 µm in (a) and 1 mm in (b–d).

Because PHP.eB‐FLEX‐tdTomato produced sparse tdTomato expression in neurons and astrocytes across the cerebral cortex, cerebellum, and other brain regions in every Cre‐driver strain we tested, we next evaluated the possibility that Cre‐independent recombination of the FLEX cassette was occurring (Botterill et al. 2021). We injected the same vector in three Cre‐negative mice, including one littermate from the *Foxp2*‐IRES‐Cre colony (female, injected at 36 days) and two C57BL/6J mice (male and female, injected at 29 and 28 days). All three Cre‐negative cases had scattered tdTomato expression across the brain, primarily in the cerebral cortex and in cerebellar Purkinje neurons (Figure 14a–d).

## 4 Discussion

In this study, we evaluated the performance of PHP.eB as a vector for brain‐wide delivery of Cre‐dependent genetic tools. The diverse patterns we identified in different brain regions and cell types demonstrate important differences in viral tropism and transduction efficiency, indicating that for some neuronal populations, PHP.eB is not an ideal delivery vector. We also observed scattered, Cre‐independent expression throughout the brain. These findings reveal inherent trade‐offs between broad delivery and transduction specificity that should be considered in any experimental designs that incorporate PHP.eB.

### 4.1 Theoretical Advantages of PHP.eB

AAV vectors are used widely for cell‐type‐specific neuroscience experiments because they allow efficient delivery of highly specific, Cre‐dependent genetic tools in Cre‐recombinase animals (Betley and Sternson 2011). Although the limited blood–brain barrier penetration of most AAVs hampers efficient delivery into the central nervous system (Wu et al. 2006), AAV‐PHP.eB facilitates transduction of cells within the central nervous system by crossing the blood–brain barrier after peripheral injection (Chan et al. 2017). To do this, the PHP.eB capsid requires a specific receptor, LY6A, which is present in the endothelium of cerebral blood vessels in C57BL/6J mice (Hordeaux et al. 2019; Huang et al. 2019).

PHP.eB may allow relatively selective transduction of specific cell types by using various enhancers and promoters (Ben‐Simon et al. 2025; Chan et al. 2017; Furlanis et al. 2025; Hunker et al. 2025; Radhiyanti et al. 2021). Despite the exciting therapeutic potential of this type of approach, there are few brain‐wide studies comparing transduction efficiency and possible tropism across brain regions and neuronal subtypes. Some investigators have attempted peripheral delivery of a Cre‐dependent genetic construct using PHP.eB (Claflin et al. 2022; Flippo et al. 2022), or an earlier iteration, PHP.B (Allen et al. 2017; Deverman et al. 2016). Combined with the considerable array of Cre‐driver mice currently available, this approach could be a platform for targeting and testing widespread neurons in ways that are impractical or impossible using stereotaxic injections. Moreover, even when stereotaxic AAV delivery is feasible, peripheral injection is more convenient and less invasive.

PHP.eB vectors could enable and improve neuroscience experiments in several specific ways. First, PHP.eB can be used to target widespread populations like medium‐spiny neurons throughout the striatum (Hunker et al. 2025) or inhibitory interneurons throughout the cerebral cortex (Furlanis et al. 2025), enabling approaches that would be impractical or imprecise with stereotaxic injections. Second, making accurate stereotaxic injections requires waiting typically 2 months for skull growth, followed by additional weeks of waiting for protein production and distribution, but PHP.eB performs optimally when injected shortly after weaning (Ben‐Simon et al. 2025; Furlanis et al. 2025)—this reduces the overall time each mouse is used and accelerates each experimental cohort by several weeks. Third, even when targeting a localized neuronal population that is amenable to direct AAV injection, using PHP.eB could obviate the need for stereotaxic microsurgery, which requires specialized training and equipment, takes up to an hour per mouse, and involves significant injection‐site variability. In contrast, retro‐orbital injection requires no specialized equipment, limited training, and less than a minute per animal. Thus, if certain conditions are met, PHP.eB would offer a simpler alternative with theoretically higher probability of success.

### 4.2 Critical Evaluation of PHP.eB Tropism and Specificity

These theoretical advantages depend on a key premise—that PHP.eB delivers its contents equally to all neurons in all brain regions, without selectively preferring or avoiding neuronal subtypes or brain regions. However, the present findings argue against this premise. PHP.eB transduced a minority of neurons in most brain regions, and some populations had little to no expression. For example, in *Foxp2*‐IRES‐Cre mice, very few neurons in the prominent *Foxp2*‐expressing populations of the inferior olivary nucleus and parafasicular thalamic nucleus expressed tdTomato despite robust expression in *Foxp2*‐expressing neurons in layer 6 of the cerebral cortex. Additionally, in *Hsd11b2*‐IRES‐Cre cases, zero HSD2 neurons expressed tdTomato. In *Lmx1b*‐2A‐Cre cases, a small minority of *Lmx1b*‐expressing neurons in the raphe nuclei expressed tdTomato despite robust tdTomato expression by *Lmx1b*‐expressing neurons located laterally in the trigeminal sensory nuclei. Even within the same nucleus, we found evidence of tropism inhomogeneity, exemplified by a gradient from sparse expression in *Lmx1b*‐expressing neurons in the caudal nucleus of the solitary tract to robust expression in rostral‐lateral parts of the same nucleus. These results strongly suggest a transduction bias toward some neuronal subtypes over others. Tropism was also evident in Cre‐negative mice, in which spontaneous tdTomato expression did not appear randomly or evenly but appeared more frequently in Purkinje neurons in the cerebellum and pyramidal neurons in the cerebral cortex.

### 4.3 Non‐Cre‐Dependent Expression at Brain‐Wide Scale

A key premise, often unstated, in cell‐type‐specific experiments is that expression of a recombinase‐conditional construct is limited to cells that express the recombinase‐driver gene. Cre‐negative cells should not express a Cre‐dependent construct, and in a Cre‐negative animal, no cells should express the construct. However, our experience with stereotaxic delivery of Cre‐conditional constructs has indicated that Cre‐independent expression occurs in a small minority of cells in some injection sites. Consistent with this observation, spontaneous (Cre‐independent) expression was reported in small numbers of cells after injecting AAVs with FLEX/DIO constructs into the hippocampus and neocortex of Cre‐negative mice (Botterill et al. 2021).

Whether Cre‐independent recombination of FLEX/DIO constructs occurs in vivo or during viral vector production (Matsushita et al. 2023), this limitation poses minimal concern for AAV injections targeting small neuronal populations. Even at a 0.01% nonspecific rate, an injection reaching 10,000 neurons would cause nonspecific expression in just one non‐Cre‐expressing cell. With 100 on‐target, Cre‐expressing neurons (like the HSD2 neurons in the nucleus of the solitary tract; Gasparini, Peltekian, et al. 2024), this would yield 99% transduction specificity, which is suitable for cell‐type‐specific work.

However, brain‐wide delivery changes this calculus. With ∼71 million neurons per mouse brain (Herculano‐Houzel et al. 2006), that same 0.01% rate following peripheral PHP.eB injection would transduce 7100 neurons (plus glia), reducing transduction specificity to 1.4% for the same, 100‐neuron population. Experimental impact would depend on the specific application. Chemogenetic activation with 98.6% nonspecificity may yield uninterpretable results, while recording activity in a focal population would be less affected by nonspecific expression in distant neurons. For a larger target population with on‐target expression in 100,000 neurons, nonspecific expression would be less than 5% brain‐wide. Thus, although Cre‐independent expression affects a large number of neurons brain‐wide, the experimental impact depends on the specific application, target population size, and transduction efficiency.

### 4.4 PHP.eB Transduction Efficiency and Tropism

Except for the *Hsd11b2*‐IRES‐Cre strain, peripheral injection of PHP.eB‐FLEX‐tdTomato produced a distinct tdTomato expression pattern in every Cre‐driver strain we tested. In each strain, tdTomato expression largely conformed to the distribution of Cre‐driver gene expression. However, we did not find any brain region or neuronal population in which all target neurons expressed tdTomato, and some populations had very little labeling. Expression was low in raphe *Lmx1b* neurons, yet high in nearby trigeminal nuclei, and in that same strain, expression was low in caudal yet high in rostral parts of the nucleus of the solitary tract. We detected virtually zero expression in *Foxp2* neurons in the inferior olivary nucleus yet abundant expression in *Foxp2* neurons throughout layer 6 of the cerebral cortex. Although zero *Hsd11b2* neurons expressed tdTomato, most *Nps* neurons expressed it. The reasons for such wide variance in PHP.eB transduction efficiency are unclear.

Lack of expression in the inferior olivary nucleus was particularly surprising because it is the most prominent site of *Foxp2* mRNA expression and FoxP2 protein immunoreactivity in the brainstem. Stereotaxic injection of the same vector (AAV‐PHP.eB‐FLEX‐tdTomato) directly into this region successfully transduced inferior olivary neurons, similar to a previous report of non‐Cre‐conditional PHP.eB injection directly into this region (Dorgans et al. 2022). This control experiment showed that PHP.eB can transduce inferior olivary neurons and that the neurons can express Cre and tdTomato, which leaves a deficit in viral penetration through the blood–brain barrier of this hindbrain region as the most likely explanation, even though the inferior olivary nucleus lies in a well‐perfused region of the ventral medulla, proximal to medial perforators from the basilar artery. Endothelial LY6A is necessary for PHP.eB transport across the blood–brain barrier (Hordeaux et al. 2019), but the regional distribution and density of this receptor remain poorly characterized and may contribute to observed patterns of uneven tropism.

## 5 Limitations

Several limitations should be considered when interpreting these results. First, we urge caution in extrapolating results from these four Cre‐driver strains to other Cre mice, non‐C57BL/6J‐background mice, or other species. Second, we are not aware of any known factor, such as differential membrane protein expression, that would explain the apparent regional and neuronal tropism differences observed here. Although investigating these mechanisms is beyond the scope of the present study, it could yield interesting findings. Third, while we used the same viral dose across mice, injecting more virions could transduce more neurons (Dayton et al. 2018), though it is unclear if this would affect the tropism patterns evident in our results. Our goal was to compare tropism and specificity across cell types rather than maximize transduction numbers. Fourth, we focused exclusively on the brain, so our results neither confirm nor refute prior evidence that PHP.eB additionally transduces cells in visceral organs like the liver (Brittain et al. 2025; Goertsen et al. 2022). Fifth, nonspecific expression could potentially be minimized using spacer sequences that reduce Cre‐independent FLEX recombination (Fischer et al. 2019; Matsushita et al. 2023), and using a neuron‐specific promoter like hSyn should eliminate the expression we observed in astrocytes. Sixth, neurons in the subthalamic nucleus express both *Foxp2* and *Lmx1b* yet exhibited very little tdTomato expression in *Foxp2*‐IRES‐Cre mice despite robust expression in *Lmx1b*‐2A‐Cre mice. Since viral tropism cannot explain this discrepancy in the same neuronal population, insufficient Cre protein production due to the IRES linker (relative to the P2A linker in *Lmx1b*‐2A‐Cre mice) may account for reduced expression in *Foxp2*‐IRES‐Cre mice (Hofacre et al. 2018; Kim et al. 2011).

## 6 Conclusion

Our results confirm that peripheral delivery of PHP.eB can transduce widely distributed neuronal populations, and this may be useful for some neuroscience applications. However, we found widely variable transduction efficiency across brain regions and neuronal subtypes, and these limitations necessitate first evaluating the ability of PHP.eB to transduce each neuronal population of interest before an experimental or therapeutic application. We also identified a low level of non‐Cre‐dependent expression that could present a meaningful confound for experimental approaches that use PHP.eB for Cre‐conditional targeting at brain‐wide scale. These findings reveal inherent trade‐offs between broad delivery and transduction specificity that should be considered in experimental designs that incorporate PHP.eB.

## Author Contributions

All authors contributed to the study conception and design. Material preparation, data collection, and analysis were performed by Haidong Zhu. The first draft of the manuscript was written by Haidong Zhu and Joel C. Geerling, and all authors commented on the following versions of the manuscript. All authors read and approved the final manuscript.

## Funding

This work was supported by the National Institutes of Health (NIH R01 NS130038 to J.C.G., University of Iowa); the Aging, Mind, and Brain Initiative (to J.C.G., University of Iowa); and the Early Stage Investigator Award (to J.C.G., Carver Trust and the Iowa Neuroscience Institute).

## Ethics Statement

All procedures performed in studies involving animals were in accordance with the ethical standards of the institution or practice at which the studies were conducted.

## Conflicts of Interest

The authors declare no conflicts of interest.

## Data Availability

The data that support the findings of this study are available from the corresponding author upon reasonable request.
